# Characterization of various acrylate based artificial teeth for denture fabrication

**DOI:** 10.1007/s10856-022-06645-8

**Published:** 2022-01-24

**Authors:** Nawshad Muhammad, Zenab Sarfraz, Muhammad Sohail Zafar, Saad Liaqat, Abdur Rahim, Pervaiz Ahmad, Abdullah Alsubaie, Abdulraheem S. A. Almalki, Mayeen Uddin Khandaker

**Affiliations:** 1 Department of Dental Materials, Institute of Basic Medical Sciences, Khyber Medical University, Peshawar, 26100 Pakistan; 2 Department of Dental Materials, Akhtar Saeed Medical and Dental College, Lahore, 54600 Pakistan; 3 Department of Restorative Dentistry, College of Dentistry, Taibah University, Al Madinah, Al Munawwarah 41311 Saudi Arabia; 4 Interdisciplinary Research Centre in Biomedical Materials (IRCBM) COMSATS University Islamabad, Lahore Campus, Lahore, 54600 Pakistan; 5 Department of Physics, University of Azad Jammu and Kashmir, Muzaffarabad, 13100 Pakistan; 6 Department of Physics, College of Khurma, Taif University, P.O. Box 11099, Taif, 21944 Saudi Arabia; 7 Department of Chemistry, Faculty of Science, Taif University, Taif, 21974 Saudi Arabia; 8 Center for Applied Physics and Radiation Technologies, School of Engineering and Technology, Sunway University, 47500 Bandar Sunway, Selangor Malaysia

## Abstract

Acrylic resins-based artificial teeth are frequently used for the fabrication of dentures has and contribute a very strong share in the global market. However, the scientific literature reporting the comparative analysis data of various artificial teeth is scarce. Focusing on that, the present study investigated various types of commercially available artificial teeth, composed of polymethyl methacrylate (PMMA). Artificial teeth are characterized for chemical analysis, morphological features, thermal analysis, and mechanical properties (surface hardness, compressive strength). Different types of artificial teeth showed distinct mechanical (compression strength, Vickers hardness) and thermal properties (thermal gravimetric analysis) which may be attributed to the difference in the content of PMMA and type and quantity of different fillers in their composition. Thermogravimetric analysis (TGA) results exhibited that vinyl end groups of PMMA degraded above 200 °C, whereas 340–400 °C maximum degradation temperature was measured by differential thermal analysis (DTA) for all samples. Crisma brand showed the highest compressive strength and young modulus (88.6 *MPa* and 1654 *MPa)* while the lowest value of Vickers hardness was demonstrated by Pigeon and Vital brands. Scanning electron microscope (SEM) photographs showed that Crisma, Pigeon, and Vital exhibited characteristics of a brittle fracture; however, Artis and Well bite brands contained elongated voids on their surfaces. According to the mechanical analysis and SEM data, Well bite teeth showed a significantly higher mechanical strength compared to other groups. However, no considerable difference was observed in Vickers hardness of all groups.

**Figure:**
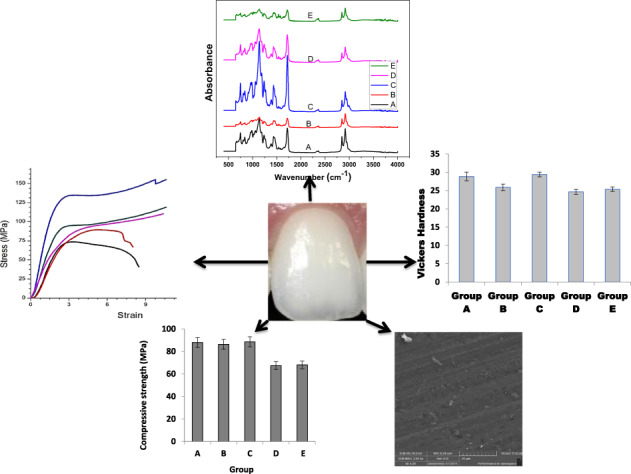
Graphical abstract

Graphical abstract

## Introduction

Acrylic resins have been used to fabricate artificial teeth for prosthetic rehabilitation of edentulous patients using removable dentures [1, 2]. Despite the advancement in the prevention and conservative treatment modalities, the demand for prosthodontic rehabilitation continues to sharply increase particularly due to the increase in the life expectancy and elderly population [3, 4]. While choosing the artificial teeth for denture fabrication, physical and mechanical properties are of the utmost importance not only for the dental team but also for the elderly patients who would be using the dentures. Ideally, artificial teeth should closely mimic the esthetic and anatomical details like the natural dentition. In addition, the material should be non-toxic, non-reactive with oral soft tissues, insoluble in oral fluids, lightweight, and cost-effective. To restore the functional capabilities, the artificial teeth should have good mechanical properties (elastic modulus, microhardness, and wear resistance) to assure longevity and withstand the masticatory forces without failure. Lack of sufficient mechanical properties would result in loss of posterior tooth support, loss of masticatory efficiency [5], loss of vertical dimension of occlusion, fatigue of masticatory muscles, alterations in the functional path of masticatory movement [6], faulty tooth relationship, and temporomandibular dysfunction. Similarly, good wetting characteristics of the artificial tooth are also important because materials with different surface energies have distinct wettability [7].

Currently, various types of artificial denture teeth including conventional and modified acrylic resin including high cross-linked acrylic resin and composite resin teeth are commercially available. Although artificial porcelain teeth had been used for many years due to their durability, poor chemical bonding to denture base and brittleness are the main issues. Moreover, there is a mismatch in the coefficient of thermal expansion of porcelain teeth with the acrylic denture base [8]. Porcelain teeth can also cause trauma to the underlying supporting soft tissues, clicking sound, and attrition of the opposing teeth

Polymethyl methacrylate (PMMA) is one example of the important unmodified acrylic materials used in dentistry. It has important applications in industrial polymeric materials and is an active material for research in various fields [9–12]. PMMA has also been used commonly as a denture base material due to its favorable properties including non-toxicity [13], good biocompatibility with oral tissues, dimensional stability, absence of taste, teeth adhesion [14], and insolubility in body fluids. Furthermore, these acrylic resin based artificial teeth have several advantages of ease of manipulation, the possibility of characterization, the glossy surface after polishing [15], color stability, [16], and good quality esthetic outcome [17–19]. However, some disadvantages of acrylic resins are also reported, for example, acrylic teeth are susceptible to abrasion and may contain microscopic pits that can hold microorganisms [20].

A few researchers also published the properties of acrylic resin based artificial teeth [21–26]. Matheus et al. [23] evaluated the composition, hardness, and wear resistance of acrylic resin artificial teeth exposed to mechanical tooth brushing. The correlation between the wear resistance, hardness, and elastic modulus of artificial denture teeth was also determined [25]. Katia et al. [25] evaluated the wear resistance of PMMA denture teeth (Trilux and Vivodent) based on their chemical composition. In a pilot study [26], the wear behavior of artificial teeth materials with variable distribution of PMMA and cross-linked PMMA fillers has been quantified.

Focusing on the current market scenario of artificial teeth, the current study provides a platform for technical information and future research gap related to PMMA based artificial teeth considering that the scientific literature reporting the comparative analysis data of various artificial teeth is scarce. The present study was aimed to analyze various types of commercially available PMMA based artificial teeth for their physical, chemical, and mechanical properties. For this purpose, we employed a range of materials characterization techniques including Fourier transform infrared spectroscopy (FTIR), thermogravimetric analysis (TGA), differential thermal analysis (DTA), mechanical testing, SEM, and water uptake behavior.

## Materials and methods

The present study included five different types of commercially available PMMA based artificial teeth (already polymerized) were purchased from the local market (Table 1). The brand names and their study group division used in the study are listed in Table 1.

**Table 1:** Brands and respective groups of selected PMMA artificial teeth

Sr. No	Brand	Group	Composition
1	Artis	A	Acrylic
2	Well bite	B	Acrylic
3	Crisma	C	Acrylic
4	Pigeon	D	Acrylic
5	Vital	E	Acrylic

### Fourier transform infrared spectroscopy (FTIR)

All groups of artificial teeth were characterized by FTIR spectroscopy (FTIR, Nicolet 6700, USA) using photoacoustic cell mode (PAC) purged with helium gas. All spectra were collected with wavenumber ranging from 4000 to 400 cm^−1^ during 256 scans and the resolution of 8 cm^−1^.

### Thermogravimetric analysis

TGA analysis was carried out by using an SDT Q 600 TGA analyzer (TA Instruments, USA). The heating rate was 10 °C min^–1^ from ambient temperature to 800 °C under nitrogen flow. The weight loss experienced by the samples as a function of temperature provided the thermal degradation behavior and rate of degradation of artificial teeth. DTA for samples was measured by taking derivatives of weight concerning temperature.

### Compression tests

Six samples of each composition were tested for compression. Tests were performed on the universal testing machine (M 500-50AT) at a crosshead speed of 1 mm/min. All samples were formed in cylinders (6 mm in diameter and 4 mmin height) according to ASTM D 695-02a (ISO 604) standard [27].

### Microhardness testing

HVS-1000 microhardness tester was used for Vickers hardness measurement. For each brand, five indentations were made in different regions of each tooth. Vickers hardness was measured by a software program connected to the hardness tester by measuring the two diagonals of the indentation left on the sample by the pyramidal diamond penetrator. A 25 g load was used, applied for 15 s. Samples were prepared according to ASTM E 384-89 standard [28]. Surfaces of the acrylic resin denture teeth were ground flat using 600, 800, 1000, and 1200 grit silicon carbide paper, and final polishing was performed with 1500 grit before the test.

### Scanning electron microscopy (SEM)

The morphology of fracture surfaces of artificial teeth brands was studied using a scanning electron microscope (TESCAN Vega 3 LMU). SEM was operated at secondary electron image mode. All images were obtained at an accelerating voltage of 20 KV. The specimens were mounted on an aluminum stud and the surfaces of samples were gold coated to eliminate the electrostatic charging effect and to enhance a better image resolution in a Quorum sputter coater.

### Water behavior

The water uptake of samples was determined according to ISO standard 4049:2000. 12 discs of 15 mm diameter and 1 mm thickness were prepared. All samples were placed in a desiccator until a constant weight was achieved. Dry weights (W_i_) were immersed in distilled water at 37 °C. The discs were removed at different periods (at day 1, 3, 5, and 7), dried weighted, and re-immersed for 1 week. The wet weights of samples were measured as W_f_. The water uptake percentage (W%) is calculated by Eq. 1:

1 $${{{\mathrm{W}}}}\% = {{{\mathrm{Wf}}}} - {{{\mathrm{Wi}}}}/{{{\mathrm{Wi}}}} \ast 100$$

### Statistical analysis

All the measurable properties were analyzed for five samples of each group and the mean data was presented in either graph along with error bars.

## Results

### FTIR

**Fig. 1:**
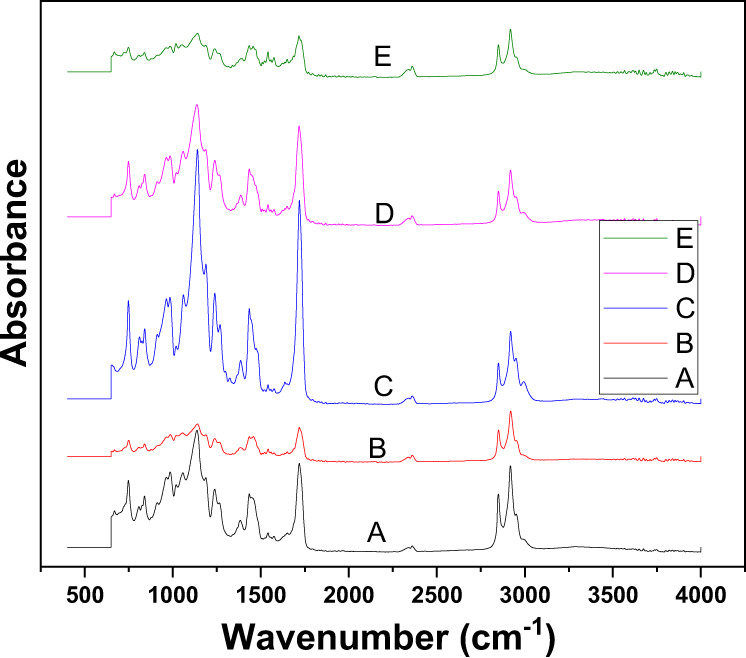
FTIR spectra of different brands of artificial teeth (Group A–E)

FTIR spectral peaks were identified for all PMMA based artificial teeth (Group A–E) shown in Fig. 1. The peaks related to C–O are appeared ranging from 1000 to 1300 cm^−1^. C–H stretching peaks are observed at 2911–2913 cm^−1^ in all samples. An intense peak was exhibited by each group at 1731 cm^−1^. Other peaks in the region 950 cm^−1^ −650 cm^−1^, 744 cm^−1^, 1429 cm^−1^, 1731 cm^−1^, 976 cm^−1^, 1046 cm^−1^, and around 800–850 cm^−1^ were observed.

### Thermal analysis

**Fig. 2:**
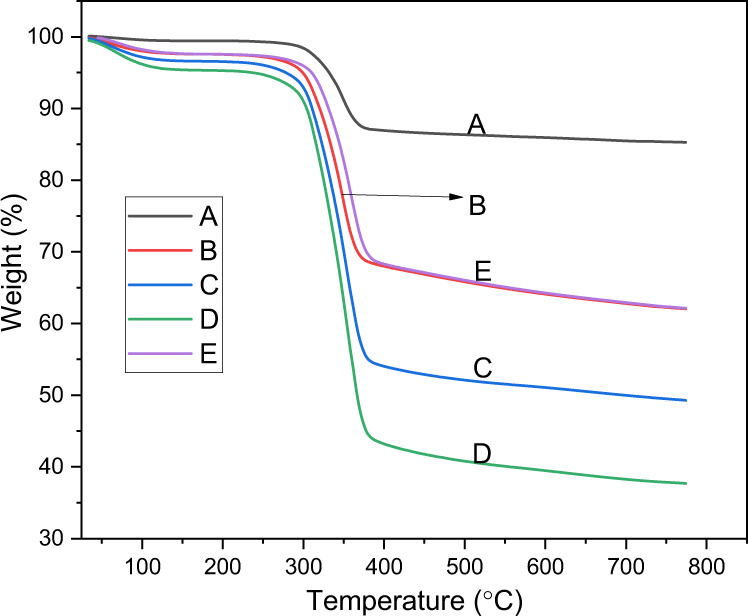
TGA curves illustrating the thermal degradation temperatures of the PMMA based artificial teeth

**Fig. 3:**
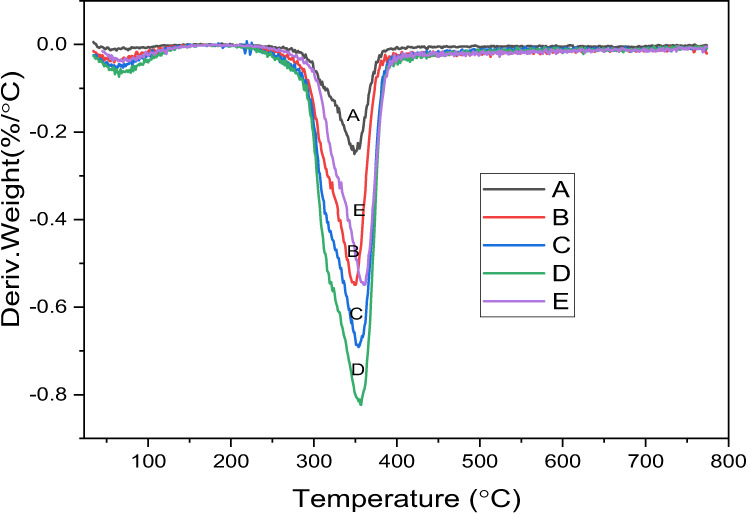
DTA of artificial teeth Group A–E

The TGA scans of samples A–E are shown in Fig. 2. The thermogram of all samples showed a three-step thermal degradation behavior. Up to a temperature of 200 °C, there was no weight loss. However, a sharp decrease in the weight of each sample was observed immediately beyond 200 °C. At a temperature of 470 °C, almost complete weight loss was observed. Figure 3 presents the DTA thermographs for groups A–E. For all brands, the maximum weight loss was observed in the temperature range of 340–470 °C.

### Compression tests

**Fig. 4:**
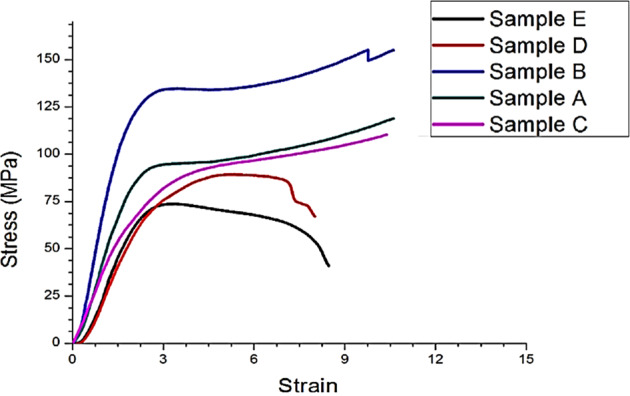
The typical stress-strain curves and values of ultimate compressive strength of the artificial teeth samples

**Fig. 5:**
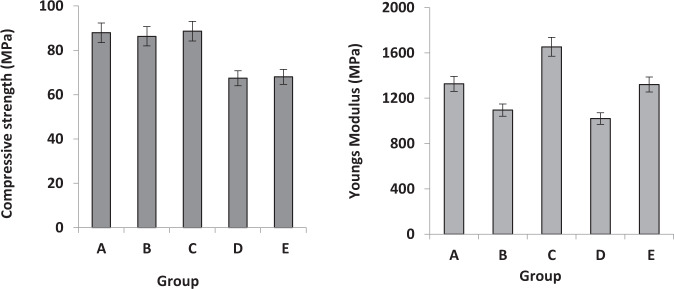
Compressive Strength (MPa) and Modulus (MPa) Data of group A–E

Figure 4 shows the compressive stress-strain curve of Group A–E. Sample B showed the highest stress/strain curve. Compressive Strength (MPa) and Modulus (MPa) Data are presented in Fig. 5 for the commercial artificial teeth, where Group C showed the maximum compressive strength and modulus of 88.6 *MPa* and 1654 *MPa*, respectively. While group E and group D showed lower values of 70–75 *MPa*.

### Vickers microhardness

**Fig. 6:**
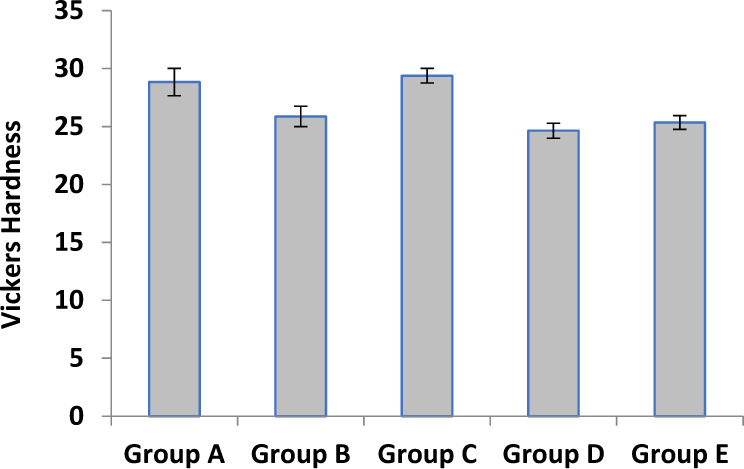
Vickers Hardness (Kg/mm^2^) Data of artificial teeth samples

Vickers hardness results of all groups of artificial teeth are shown in (Fig. 6). The values measured are 28.8, 25.8, 29.3, 24.6, and 25.3 Kg/mm^2^ for groups A, B, C, D, and E respectively.

### Scanning electron microscopy

**Fig. 7:**
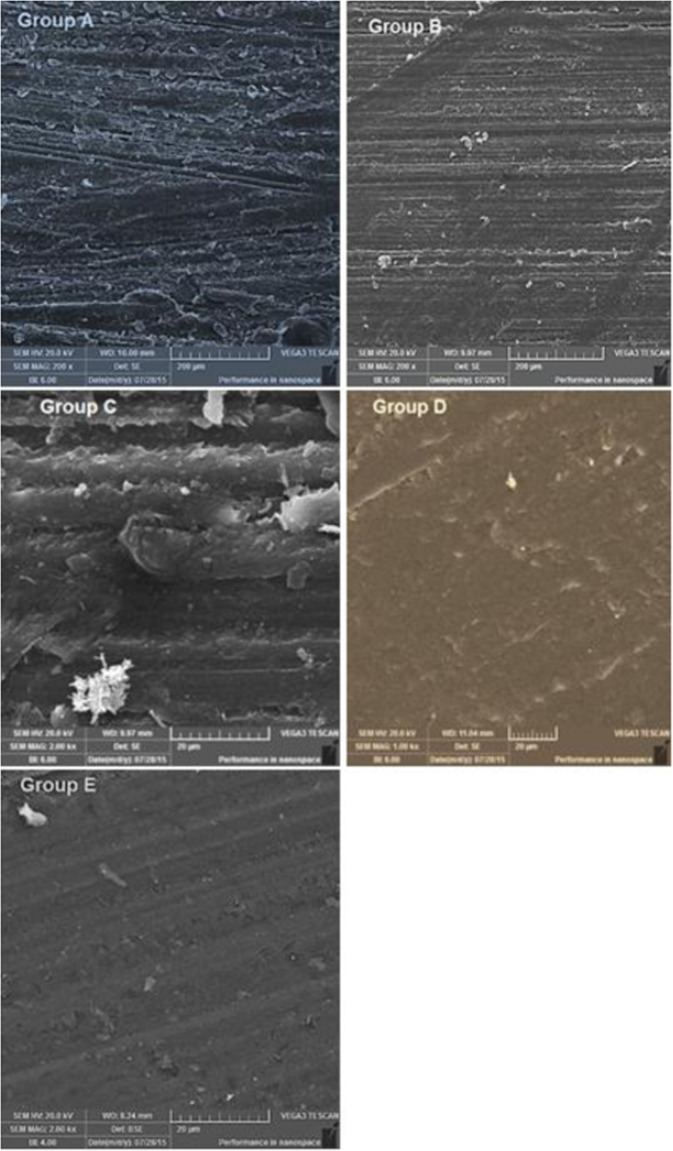
SEM micrographs of fracture surfaces of artificial teeth after compressions tests

Figure 7 shows the SEM micrographs of fracture surfaces of artificial teeth after compressions tests. Micrographs showed that Group D and E have a relatively smooth fracture section as compared to other compositions.

### Water uptake behavior

**Fig. 8:**
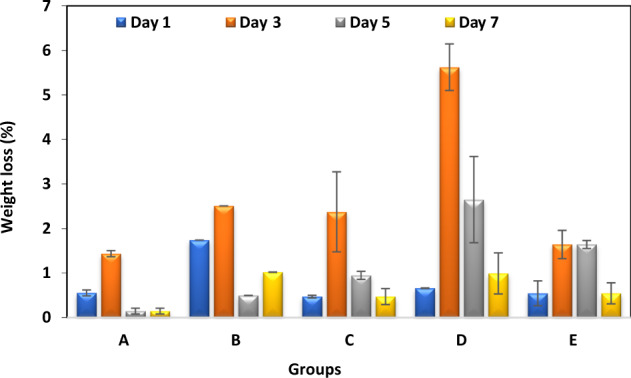
Water uptake behavior of different groups of artificial teeth with standard deviations error bars

The water uptake behavior of artificial teeth groups is shown in Fig. 8. It is clear from the figure that on day 2, all groups attain maximum water uptake. This initial mass uptake of samples in 1 day is followed by mass reduction, thereby decreasing the swelling percentage over the remaining period of day 3 and day 7.

## Discussions

The present study investigated the physical, chemical, and mechanical characteristics of various types of commercially available PMMA artificial teeth for dental prostheses. For this purpose, a range of materials characterization techniques including SEM, FTIR, TGA, DTA, water sorption, and mechanical testing was used. Due to an increase in the life expectancy and need for dental prostheses, PMMA artificial teeth are fabricated and supplied by several manufacturers to fulfill the sharply increasing global demands. The present study reported significant differences in certain physical and mechanical properties of PMMA artificial teeth fabricated by various manufacturers, which may affect the fabrication and clinical performance of dental prostheses. The FTIR is a suitable technique to identify chemical and structural changes in PMMA materials [29]. Although the FTIR spectra of all PMMA based artificial teeth demonstrated no significant difference in the entire range 400–4000 cm^−1^, the intensity variations of the bands were observed (Fig. 1). The broad peak ranging from 1000 to 1300 cm^−1^ appeared due to C–O (ester bond) stretching vibrations [30]. Another sharp and intense peak was observed at 1731 cm^−1^ which is attributed to the acrylate carboxyl group. The broad bands in all samples in the region 950 cm^−1^–650 cm^−1^ appeared due to C–H bending [23]. Furthermore, a distinct peak at around 1100–1200 cm^−1^ is attributed to the C–O–C stretching vibration. Peaks corresponding to aliphatic C–H stretch were found at 2911–2913 cm^−1^ in all samples. These findings are nearly consistent with a previous study that used FTIR to characterize the PMMA denture base materials [29]. It is clear from comparative IR spectra of artificial PMMA tooth that the intensities at 744 cm^−1^ (α-methyl group vibrations), 1134 cm^−1^ (ester bond), 1429 cm^−1^ (bending vibration of the C–H bonds of the –CH_3_ group), and 1731 cm^−1^ (acrylate carboxyl group) are relatively high for group C while group A, B, and E showed the lower intensities with almost similar values, which may be attributed to the organic (PMMA) content present in each composition of Group A–E. The bands at 976 cm^−1^, 1046 cm^−1^, and around 800–850 cm^−1^ represent the characteristic absorptions of PMMA. The above discussion demonstrated that the main structural component of all the five brands of artificial teeth is the PMMA polymer [29, 31].

The TGA is frequently used to analyze the decomposition properties of various biomaterials including PMMA [32, 33]. The TGA and DTA demonstrated a rapid increase in the weight loss or degradation ratio above 200 °C due to the existence of vinyl end groups in the polymer chains of PMMA [34], which unzip the polymeric chains through a chain transfer process. A previous study by Hay and Kemmish exhibited a weaker bonding in the end groups compared to the polymer chains structure and begin to degrade at ~220 °C [35]. As the temperature rises above 300 °C, random scission constitutes the mechanism of degradation and the depolymerization rate increases due to main chain scission [34]. The present study demonstrated a similar thermal behavior for all groups however, the amount of the final residues varied among groups. The deference in the residues might be attributed to either the proportion of inorganic fillers or related to the curing of the samples. These findings suggested that manufactures have added different types and proportions of inorganic fillers to PMMA artificial teeth to control their properties. Inorganic fillers are commonly added to the PMMA materials to enhance the mechanical and thermal properties such as flexural strength, and thermal diffusivity [36]. Therefore, the degradation characteristics of PMMA-inorganics fillers are affected by filler types and contents and have been extensively investigated [37]. Overall, no considerable difference was found in the weight loss values of each tooth. The maximum weight loss was observed in the temperature range of 340–470 °C suggesting high thermal stability at the temperature of oral cavity.

The artificial teeth are desired to have good mechanical properties to withstand the forces of mastication in the oral cavity. In terms of mechanical properties, the initial stage linear elastic region was observed which ends when the cracking of sample struts accrued (maximum compressive strength) [38]. The elastic region is followed by a collapse-plateau characteristic of brittle fracture. The maximum compressive strength and modulus of elasticity of 88.6 *MPa* and 1654 *MPa*, respectively (group C), while group E and group D showed significantly lower compressive strength and modulus of elasticity (Fig. 5). The strength values for each type are affected by several factors including chemical composition and more specifically the content and the type of inorganic fillers present in the commercial composition. The hardness of acrylic teeth is another important parameter that determines their wear behavior in the oral cavity [39]. The hardness of acrylic teeth should be enough to bear the cyclic masticatory forces and reduce the wear due to attrition and abrasion. In the present study, we compared the hardness of various acrylic teeth using Vickers hardness. The Vickers hardness of various acrylic teeth ranged from 25 to 29 Kg/mm^2^ without any significant differences. The findings are consistent with a similar study that reported Vicker’s hardness of various acrylic teeth in the range of 28–39 Kg/mm^2^ [39]. Although the hardness of PMMA teeth is significantly lower than porcelain teeth [40, 41], they do not produce clicking sounds while functioning and are least damaging to the opposing teeth. Ideally, it is desired to closely match the hardness and wear behavior of artificial teeth and natural tooth enamel. A gross mismatch in the hardness of artificial and natural teeth is likely to cause excessive wear of the softer materials (acrylic teeth). In addition, the insignificant variation in the microhardness of various acrylic teeth may be attributed to the compositional (such as inorganic fillers type, proportion) and manufacturing techniques (such as curing methods, surface treatment) [42].

The topography analysis suggested a brittle fracture corresponding to the smooth surface of all the types due to the rapid propagation of cracks. In groups A and B however, elongated voids on the surface can be observed corresponding to the ductile cracks in their SEM analysis. These results define the mechanical behavior of the materials by measuring their ability to withstand the presence of notches and crack propagation which are the important factors affecting the performance of teeth. The ductile fracture acrylic teeth provide elasticity and sufficient mechanical strength to withstand the stresses thereby resisting the cracks propagation [43, 44].

Water sorption of artificial teeth is supposed to follow Fickian diffusion kinetics [45]. It attains maximum water uptake on day 3 followed by reversing the trend. Therefore, it can be expected that a typical artificial tooth composition containing polymers (PMMA) and some inorganic fillers becomes saturated at a specific time when placed in water. In terms of composition, Group D displayed maximum water uptake of 1% within 7 days, which may be attributed to the comparatively higher proportion of organic matrix (the lowest proportion of final residues among groups) that is consistent with the TGA results as discussed earlier. In contrast, Group A showed the minimum capacity of water sorption during the whole incubation period in water. Such differences in water uptake might be related to porosity and fillers’ nature and content of the sample.

The present study provided a detailed comparison of properties of various PMMA based artificial teeth that can direct further technical information and future research for their applications in dentistry. Despite, all the artificial teeth being manufactured using PMMA, their properties vary remarkably due to either compositional or manufacturing differences and require further investigations. There are a few limitations of this study. We investigated unused teeth manufactured by different companies, so there may be differences in the manufacturing methods (such as polymerization, and compositions) that we could not associate with the data presented. In addition, this is an in vitro study that characterized artificial teeth that were not exposed to the complex oral environment. Therefore, the present study could not address various aging related effects on the artificial teeth such as how the forces of mastication, cyclic loading, wear behavior, pH, and temperature changes may affect the physical and mechanical properties.

## Conclusion

The present study provides a comprehensive comparative analysis of different types of commercially available teeth for denture fabrication. The chemical characterization through FTIR indicated the presence of typical PMMA peaks of the ester bond, carbonyl group, and C–H bending. The TGA results indicate the weight degradation behavior of all groups with no significant differences in the degradation temperature. The results of mechanical tests suggest that the Well bite teeth showed a significantly higher Compressive Strength (88.6 MPa) and Modulus (1654 MPa). Vickers hardness were measured 28.8, 25.8, 29.3, 24.6, and 25.3 Kg/mm^2^ for groups A, B, C, D, and E respectively. Pigeon-type teeth have shown higher uptake of water. Moreover, all types of teeth observed higher uptake of water on the 3rd day which has been subsequently reduced on days 5 and 7. To investigate the effects of a dynamic oral environment on the properties of artificial teeth, further studies are required using the simulated oral environment for aging.
